# Innovative Natural Ingredients-Based Multiple Emulsions: The Effect on Human Skin Moisture, Sebum Content, Pore Size and Pigmentation

**DOI:** 10.3390/molecules23061428

**Published:** 2018-06-12

**Authors:** Ugne Cizauskaite, Jurga Bernatoniene

**Affiliations:** 1 Institute of Pharmaceutical Technology, Lithuanian University of Health Sciences, Medical Academy, Sukileliu pr. 13, LT-50162 Kaunas, Lithuania; jurga.bernatoniene@lsmuni.lt; 2 Department of Drug Technology and Social Pharmacy, Lithuanian University of Health Sciences, Medical Academy, Sukileliu pr. 13, LT-50162 Kaunas, Lithuania

**Keywords:** multiple emulsion, *Rosmarinus officinalis*, *Avena sativa*, *Linum usitatissimum*, skin moisture, pigmentation, pore size

## Abstract

The increased interest in natural cosmetics has resulted in a higher market demand for preservative-free products based on herbal ingredients. An innovative W/O/W type emulsions containing herbal extracts were prepared directly; its cation form was induced by an ethanolic rosemary extract and stabilized using weak herbal gels. Due to the wide phytochemical composition of herbal extracts and the presence of alcohol in the emulsion system, which can cause skin irritation, sensitization or dryness when applied topically, the safety of the investigated drug delivery system is necessary. The aim of our study was to estimate the potential of W/O/W emulsions based on natural ingredients for skin irritation and phototoxicity using reconstructed 3D epidermis models in vitro and to evaluate in vivo its effect on human skin moisture, sebum content and pigmentation by biomedical examination using a dermatoscopic camera and corneometer. According to the results obtained after in vitro cell viability test the investigated emulsion was neither irritant nor phototoxic to human skin keratinocytes. W/O/W emulsion did not cause skin dryness in vivo, despite the fact that it contained ethanol. We can conclude that the emulsion is safe for use as a leave-on product due to the positive effect on human skin characteristics or as a semisolid pharmaceutical base where active compounds could be encapsulated.

## 1. Introduction

In recent years, the interest in development of natural ingredient-based topically applied cosmetics and pharmaceuticals has grown [1]. According to the studies of Zhu and Woerdenbag and Choochote et al., the application of bioactive phytochemicals from herbal extracts is a potential alternative to synthetic substances to increase therapeutic effectiveness, minimize toxicity or side-effects and ensure environmental safety due to the biodegradability to nontoxic compounds [2,3]. However, it has been reported that some of the natural ingredients widely used in cosmetics and pharmaceutical preparations such as argan oil, *Arnica montana* extract, lavender, peppermint and tea tree essential oils, etc. can cause contact dermatitis and urticaria [4,5,6]. Crop (oat, rice, whey) proteins also may induce an immune-mediated allergic reaction [7,8,9]. Therefore the safety assessment of topically applied pharmaceuticals and cosmetics containing known sensitizing agents must be carried out so the toxicological evaluation of any created W/O/W systems is mandatory. 

The investigated multiple water in oil in water (W/O/W) emulsion containing herbal extracts was prepared directly by an innovative technique—the multiple emulsion formation was induced by the ethanolic rosemary extract and its active ingredients [10]. Such an approach simplifies the manufacturing process by allowing skipping all technological stages other than a two-step or phase inversion technique, is less time consuming and doesn’t require any additional surfactants [11,12,13]. The stability of the innovative W/O/W emulsion was ensured by using weak linseed and oat gels containing polysaccharides [11,14,15]. According to Fuchs et al. rosemary-containing cream has a potential protective effect against sodium-lauryl-sulfate (SLS)-induced irritant contact dermatitis; however, due to the wide phytochemical composition of rosemary, linseed and oat extracts used to form and stabilize the multiple emulsion, further in vitro and in vivo studies must be carried out to ensure the safety of the investigated sample [16].

To evaluate the influence of natural ingredients-based W/O/W type emulsion on human skin in vivo, it is necessary not only to confirm the toxicological data obtained in the in vitro study but also to investigate the effects of the innovative system on human skin characteristics such as moisture, sebum content and pigmentation. According to the data found in the literature, the use of ethanol- containing cosmetics, disinfectants and personal hygiene products results in skin moisture loss and skin dryness [17]. Since the investigated semi-solid drug delivery system contains an ethanolic rosemary extract, further studies must be carried out to evaluate any possible negative effects on human skin. However, Rasul and Ahtar have determined that the use of emulsions containing plant extracts of the *Lamiaceae* family, which have strong antioxidant effects, results in a significantly reduced transepidermal water loss after 2 weeks [18]. Another study determined that daily (7 days) use of an aqueous gel containing 3% of rosemary essential oil resulted in a skin moisture increase by 4% [19]. Park et al. have shown that carnosic acid inhibited matrix metalloproteinases induced by UVA in addition to UVB in human dermal fibroblasts. Carnosic acid also lessened UVB induced ROS generation [20]. 

According to Vie et al., *Avena sativa* extracts are able to modulate SLS-induced skin irritation, confirming their preventive effects on alteration of the cutaneous barrier function and microvasculature [21]. These data correspond with other studies, which have determined that the topical three-week application of oat extract containing β-glucans significantly increased moisture content and improved skin barrier function [22]. Criquet et al. have determined that hydration of the forearm skin increased significantly (5.6–22.2%) during the period (days 1–28) of oatmeal-containing cream application and afterwards (day 42) compared with baseline. In addition it has been stated that oatmeal-containing personal care products have a very low irritantion potential as well as a very low allergen sensitization potential—low-level reactions were documented only in 1.0% of subjects [23]. According to Ayurvedic literature, linseed is believed to control aging processes, improve wound healing and improve the moisture holding capacity of the skin [24]. It is also known that oligo- and polysaccharides present in oats and linseed slow down skin aging by stimulating cell proliferation of cultured human skin fibroblasts, protecting cells against ascorbate-induced cytotoxicity due to the release of reactive oxygen species [25]. However, to our knowledge, the impact of multiple emulsions containing linseed and oat extract on human skin characteristics has never been investigated.

The aim of our study was to evaluate the effect of natural ingredients-based preservative free multiple emulsions on human skin moisture, sebum content and pigmentation by performing in vivo biomedical examinations. The results of the research would ensure the safety of the product and its possible commercialization as a skin care product or pharmaceutical base, to which lipophilic and hydrophilic compounds can be added.

## 2. Results

To evaluate the safety of topically applied pharmaceuticals or cosmetics and investigate their effect on human skin in vivo, the composition of the product itself must be taken into consideration. Due to the wide phytochemical composition range of herbal extracts which were used to create and stabilize multiple W/O/W type emulsion, it is relevant to determine the concentration of active ingredients contained in the semi-solid emulsion after the preparation is finished. Obtained results are presented in Table 1. The yields of investigated acids, monosaccharides and β-glucans in the rosemary, linseed and oat extracts were determined in our previous studies [10,26]. Unfortunately, even if the yield of galactose in linseed extract was determined to be 50.72 ± 0.0004 mg/g and it was identified in the prepared multiple emulsion, its amount was too low to be quantified [11].

**Figure 1:**
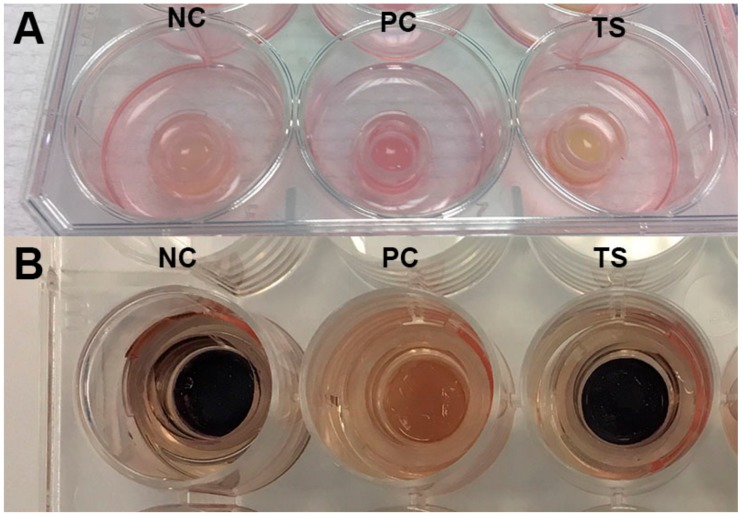
The view of 3D reconstructed human skin inserts during in vitro skin irritation evaluation before (**A**) and after (**B**) cell viability test after 18 h of incubation. NC—negative control (distilled water); PC—positive control (1% Triton X-100); TS—test sample (multiple emulsion).

Further on, the safety of the multiple emulsion containing plant extracts and ethanol had to be evaluated in vitro before the biomedical trials with human volunteers could begin. The EU Cosmetics Regulation (EC 1223/2009) foresaw a full marketing ban in Europe in 2013 for cosmetic products and ingredients tested on animals. Since then, toxicokinetics and in vitro testing were significantly improved to become a key to the integrated toxicity risk assessment based primarily on non-animal approaches [27,28,29,30]. It has brought new trends for regulations, skin tests and natural ingredients in cosmetics [31]. 3D reconstructed human epidermis models histologically matching human epidermis in vivo was a chosen assay to evaluate irritation, photo-toxicity and inflammatory effect of the investigated multiple emulsion containing herbal extracts on human skin keratinocytes. It is known that topically applied ethanol, various plant extracts and certain emulsifiers can cause skin irritation [17,32,33]. Guin has evaluated *Rosmarinus officinalis* leaf extract in a patch test with contact dermatitis or eczema—of the 234 subjects tested no subjects had a +++ reaction, though there were 16 patients with mild skin irritation [34]. Therefore the 3D reconstructed human epidermis approach was chosen to identify whether or not the created W/O/W system has irritant, phototoxic and inflammatory effects on human skin keratinocytes. According to the results obtained after cell viability test the investigated emulsion is neither irritant nor phototoxic to the skin tissue (Figure 1).

The viability of cells with topically applied emulsion did not significantly differ from the negative control. While determining the inflammatory effect, the assay media IL-1α concentration of tissues with W/O/W emulsion varied between 17–35 pg/mL. Since the concentration was below 85 pg/mL, we can conclude that multiple emulsion doesn’t cause skin inflammation in vitro [35]. Frerreira et al. have investigated the toxicity of emulsion composed of almond oil, natural antioxidant extract from grape seeds and glycolipopeptide biosurfactant on the mouse fibroblast cell line; their results indicated that emulsions containing 5 g/L of biosurfactant showed cell proliferation values of 97% [36]. Later on the effect of W/O/W emulsion based on natural ingredients on the human skin moisture, sebum content, pore size and pigmentation was evaluated by non-invasive techniques. The skin parameters were evaluated before the tests and at 15, 30, 60, 120, 180 and 240 min after product application and 7, 14, 21 and 28 days of daily use of the emulsion system. O/W type emulsions with water or water/ethanol mixture as continuous phase were used as control (Table 2).

**Figure 2:**
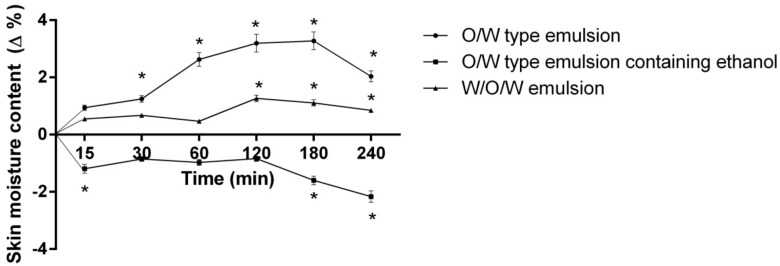
The influence of the investigated emulsions on the skin moisture content within 240 min. * *p* < 0.01 vs. 0 min. N = 51 volunteers.

Water plays a crucial role in respect to the normal function of the epidermal barrier and mechanical properties of the skin. *Stratum corneum* water content is therefore of particular interest in most topically applied cosmetics and pharmaceutics [37]. When evaluating the short-term (240 min) skin-moisturizing effect of emulsion systems, the skin moisture significantly changed after 0 to 240 min of product application depending on the emulsion system that had been used (Figure 2).

It was noted that application of the control emulsion containing ethanol results in decreased skin moisture content (by 1.6–2.16%) after 180–240 minutes (*p* < 0.05 vs. O/W emulsion and W/O/W emulsion). The moisturizing effect of control emulsion occurred after 30 min (1.25%), though three times higher moisture content in the volunteers’ skin was determined after 180 min (*p* < 0.0.1). A negative (−1.28%) skin moisture change was determined after 4 h (*p* < 0.01 vs. 180 min). Although other studies indicate that oat extract significantly affects skin moisture and improves barrier function (after 21 days of daily use skin moisture increased three times), the tested W/O/W type emulsion containing herbal extracts showed a significantly lower skin-moisturizing effect after 15–240 min compared to O/W type control emulsion [22]. Also there is a chance that such effect occurred due to the presence of ethanol in the system. It is known that application of cosmetics containing alcohol might cause skin dryness, though due to the composition of the multiple emulsion it is lower that O/W type emulsion containing ethanol [17]. The current results are in contradiction with Otto and co-authors’ assertion that the bioavailability of the multiple emulsion is higher than that of binary emulsions, which makes them more effective in moisturizing the skin [38]. 

**Figure 3:**
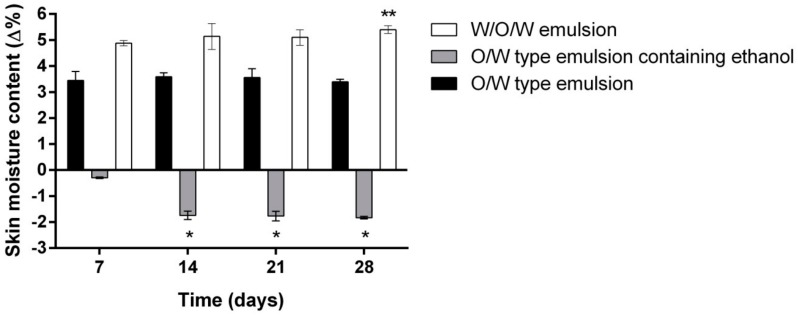
The influence of the investigated emulsions on the skin moisture within 28 days. * *p* < 0.01 vs. O/W emulsion and W/O/W emulsion; ** *p* < 0.01 vs. 7 days. N = 42 volunteers.

**Figure 4:**
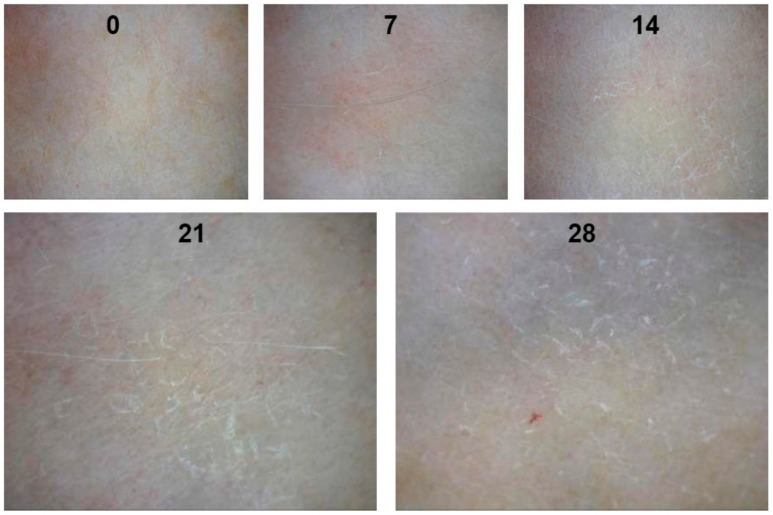
The effect of the O/W type emulsion containing ethanol on the skin condition after 0, 7, 14, 21 and 28 days. N = 42 volunteers.

In the next stage of the study, the long-term moisturizing effect was evaluated. The participants applied the given emulsion samples daily on the inner forearm for 28 days. A significant difference in skin moisture was found among the tested emulsions (Figure 3). As it was stated above, after 240 min of application the moisturizing effect of O/W emulsion was 1.19% higher than that of the multiple emulsion; however, the opposite effect was observed after a long time (28 days) use: the moisture content in the human skin was from 1.43 to 2.11% higher while using W/O/W emulsion (*p* < 0.01 vs. O/W type emulsion). It has been reported that emulsions containing plant extracts from the *Lamiaceae* family significantly reduced the transepidermal water loss after 2 weeks of daily use [18]. The skin moisturizing effect of the control emulsion did not change significantly during the 28-day period. The control emulsion containing ethanol (90%) has been shown to have a drying effect, which increased by five times after applying the product daily for 2 weeks or longer (*p* < 0.01 vs. 7 days) (Figure 4). The results agreed with the data obtained by other scientists: the presence of ethanol in the cosmetics or topically applied pharmaceutics may cause skin irritation and dryness [39,40,41].

According to the obtained results, the W/O/W type emulsion containing herbal extracts does not cause skin dryness, although it contains the same ethanol concentration as the O/W type control emulsion with ethanol. 

In assessing the effect of investigated emulsion on the skin pore size it was found that the average pore size did not differ significantly among the tested samples (Table 3). Flament et al. have stated that the average pore size is 40–80 μm depending on the age and genetics of the subject [42]. It was observed that the pore size of subjects who used the O/W type emulsion containing ethanol increased by 7.28–15.89% after 7–28 days (*p* < 0.01). According to other studies, the skin pore size increases with age, elevated lipid excretion and rough, dry skin conditions [43,44]. No significant changes in skin pore size has been found in other investigated groups. 

**Figure 5:**
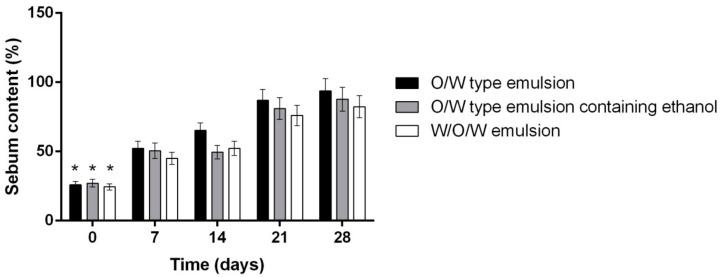
The influence of investigated emulsion on the skins sebum content. * *p* < 0.05 vs. 7, 14, 21, 28 days. N = 42 volunteers.

The amount of sebum at the surface of the skin increased significantly after 7 days of daily use of the tested emulsion systems. In the biomedical trial it was determined that the lipid content of the skin after 28 days of daily use of multiple emulsion was 6.76% higher than the O/W emulsion and 13.28% higher than O/W emulsion containing ethanol (*p* < 0.01). We hypothesized that such effect could occur due to the presence of alcohol in the emulsion system, though Kramer et al. have determined that daily use of disinfectants containing more than 60% of alcohol has no significant effect on superficial sebum content [45]. Yet according to Ahtar et al., sebum content in the skin depends only on the lipophilic phase concentration in the emulsion system [46]. Based on the data presented in Figure 3 and Figure 5 the direct correlation between the moisture and sebum content was determined (corr. coefficient 0,897).

**Figure 6:**
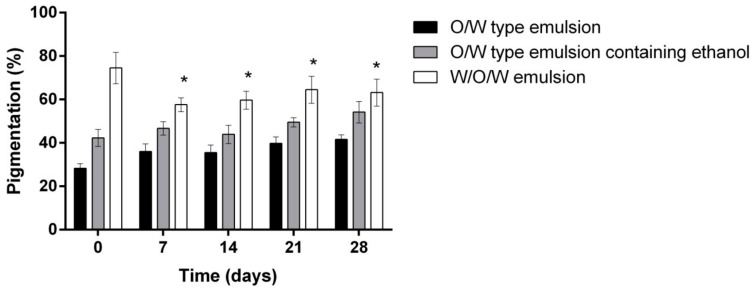
The influence of the investigated emulsions on the skin pigmentation * *p* < 0.01 vs. 0 days. N = 42 volunteers.

Further on, the influence of control emulsions and the investigated W/O/W emulsion system on the skin pigmentation was evaluated (Figure 6). As a result of the changes in the UV index (from 2 to 7) during the study, a hypothesis was raised that pigmentation in the volunteers’ skin should increase. The literature states that UV radiation promotes melanin production and skin pigmentation: melanin-containing melanosomes move closer to the surface of keratinocytes and form a cap above the nucleus to protect it from the UV damage [47,48]. The results of the hypothesis were confirmed—after 28 days the pigmentation increased by 13.39% using the control emulsion and 11.84% using the emulsion containing ethanol (*p* < 0.01). 

There was no significant difference between the control emulsions. When evaluating the effect of multiple emulsion on pigmentation, it was observed that after 7 days of the product use it decreased by 16.94% (*p* < 0.01) but after 14–28 days, with an increase in the UV index, the skin pigmentation of the subjects who used W/O/W emulsion with herbal extracts increased by 5.6% (*p* < 0.01 vs. 7 days). The potential effect of protection against UV rays may be associated with strong antioxidant and photoprotective properties of rosemary extract [49,50,51]. It is known that UV causes photoaging via the upregulation of matrix metalloproteinases [52]. A study by Offord et al. examined UVA-exposed human dermal fibroblasts and carnosic acid, a rosemary polyphenol. UVA exposure increased metalloproteinase 1 mRNA by 10–15 times. This was suppressed by carnosic acid [53]. Further studies are needed to evaluate the photoprotective properties of the investigated herbal materials.

## 3. Materials and Methods

### 3.1. Materials

Dried rosemary (*Rosmarinus officinalis* L.) leaves were purchased from UAB “Sirdazole” (Vilnius, Lithuania). Linseed (*Linum usitatissimum* Linn.) was procured from UAB “Herba humana” (Vilnius, Lithuania), oat (*Avena sativa* L.) groats were purchased from UAB “Skaneja” (Vilnius, Lithuania).

Extra virgin olive oil was purchased from UAB “Anira” (Kaunas, Lithuania). The emulsifier/rheology modifier ViscOptima™ SE (Sodium Polyacrylate, Ethylhexyl Cocoate, PPG-3 Benzyl Ether Myristate and Polysorbate 20) was provided by CRODA (Goole, UK).

HPLC and TLC standards of triterpenic acids (ursolic acid (>98%), oleanolic acid (>97%), galacturonic acid (>97%), xylose (>99%), galactose (>99%), rhamnose (>99%) and arabinose (>98%) were purchased from Sigma-Aldrich (St. Louis, MO, USA). Rosmarinic acid was obtained from ChromaDex (Santa Ana, CA, USA). Triethanolamine (>99%) was procured from Sigma-Aldrich. n-1-(-1-naphtyl)-ethylenediamine dihydrochloride (>98%) was purchased from Tokyo Chemical Industry (Tokyo, Japan).

The EpiDerm™ (EPI-200) reconstructed human skin models and MTT kit to test cell viability was purchased from MatTek (Bratislava, Slovakia). The Quantikine^®^ human IL-1α/IL-1F immunoassay was from R&D Systems (Minneapolis, MN, USA).

### 3.2. Preparation of Rosemary, Linseed and Oat Extracts

Ultrasound assisted extraction of dried rosemary leaves has been carried out in an ultrasonic bath (Bandelin Electronic GmbH & Co. KG, Berlin, Germany) with an ultrasonic peak output of 200 W, equipped with digital temperature controller/indicator. Extraction parameters were as follows: solvent:material ratio 1:15, extraction temperature 60 °C, extraction time 10 min. Ethanol (90%) was used as the solvent. To obtain viscous extracts the extraction of oats and linseed has been carried out using magnetic stirrer (MSH-20A, Witeg Labotechnik GmbH, Wertheim, Germany). Whole oat groats and linseed were extracted in 250 mL flask for 1 hour at 98 ± 2 °C temperature. The solvent:material ratio was 1:20, distilled water was chosen as the extraction solvent. The hot extracts were filtered through four layers of cheesecloth and allowed to cool to room temperature.

### 3.3. Preparation of Emulsion

The multiple and double emulsions were prepared directly by mixing 0.5% of emulsifier (sodium polyacrylate (and) ethylhexyl cocoate (and) PPG-3 benzyl ether myristate (and) polysorbate 20) into the olive oil (20%), then the continuous phase containing plant extracts/water/ethanol was added. The composition of W/O/W emulsion was optimized in our previous study according to experimental mixture design matrix using Design Expert (version 9.0.4.01, Stat-Ease Inc., Minneapolis, MN, USA) [11]. Compositions of double and multiple emulsions are presented in Table 2. Emulsions were stirred with an Eurostar 200 digital mechanical stirrer (IKA^®^-Werke GmbH & Co. KG, Staufen, Germany). The optimal technological parameters were previously determined: stirring speed 800 rpm/min and stirring time 15 min [26].

### 3.4. HPLC Conditions for Determination of Rosmarinic Acid (RA), Ursolic Acid (UA) and Oleanolic Acid (OA)

HPLC analyses have been carried out using a Waters 2695 chromatography system (Waters, Milford, MA, USA). It was equipped with a Waters 996 PDA detector. Data were collected and analyzed using the Empower2 chromatography manager software (Waters). 

The emulsion was mixed with 90% ethanol (*v*/*v*) in ratio 1:1. It was vortexed for 5 min at 2200 rpm then filtered through 0.2 µm membrane filter for HPLC analysis.

The quantity of investigated acids was determined using an ACE 5 C18 250 × 4.6 mm column (Advanced Chromatography Technologies, Aberdeen, Scotland). The mobile phase for RA determination consisted of solvent A (methanol) and solvent B (0.5% (*v*/*v*) acetic acid in water). The linear gradient elution profile was as follows: 95% A/5% B, 0 min; 40% A/60% B, 40 min; 10% A/90% B, 41–55 min; 95% A/5% B, 56 min. The flow rate was 1 mL/min and injection volume was 10 µl. Absorption was measured at 329 nm. The mobile phase for OA and UA determination was composed of methanol and water (90/10, *v*/*v*). The flow rate was 0.6 mL/min and injection volume was 10 µL. Absorption was measured at 203 nm. Quantification was carried out by the external standard method. The calibration curves were constructed (RA R^2^ = 0.999918, OA R^2^ = 0.999383, UA R^2^ = 0.998872), the peak areas were used for quantification.

### 3.5. Determination of β-glucans

The level of β-d-glucan was determined using Mixed-linkage β-glucan assay kit (Megazyme, Wicklow, Ireland) based on the method published by McCleary and Codd [54]. The absorbance measured at 510 nm against a reagent blank. The values of the β-d-glucan levels were achieved for each sample as the mean ± standard deviation. The β-d-glucan content was calculated in dry weights.

### 3.6. Determination of Monosaccharides

The preparation and hydrolysis of the samples was carried out according to a slightly modified method of Emaga et al. [55]. Fifty mg of dried linseed and oat extracts were hydrolyzed with 2 M H_2_SO_4_ (2.5 mL) at 80 °C for 2 h. The reaction medium was adjusted to pH 7 using BaCO_3_ powder. The BaSO_4_ salt was removed by centrifugation at 3000 rpm for 10 min, then the remaining solution was filtered and dried at 50 °C at 200 mb in vacuum drying oven VO200 (Memmert, GmbH & Co. KG, Schwabach, Germany). The syrups were dissolved in distilled water.

The quantitative and qualitative evaluation of monosaccharides was carried out by thin layer chromatography (TLC). The experiment was conducted on TLC Silica gel 60 F254 plates 100 × 100 mm (Merck Millipore, Burlington, MA, USA). l-Rhamnose, l-(+)-arabinose, d-(+)-galactose, d-(+)-xylose and d-(+)-galacturonic acid standards were dissolved in water, 10 µg of each standard solution and 5 µg of investigated extracts were spotted on a silica plate. The calibration curves were obtained and the calculations have been made by TLC Visualizer (CAMAG, Muttenz, Swizerland) according to the areas of peaks. The solvent system consisted of n-propanol, water, trimethylamine and 25% NH_3_ in ratio 80:20:0.2:4 respectively. Sugars were detected after spaying with about 5 mL of a solution of 6.5 mM n-1-(-1-naphtyl)ethylenediamine dihydrochloride in methanol and placing in a 100 °C oven for approximately 10–15 min [55]. The monosaccharides content was calculated in dry weights.

### 3.7. Skin Irritation, Phototoxicity and Inflammation Determination in Vitro

Skin irritation in vitro was determined using EPI-200 skin inserts according to OECS TG 439 guidelines and validated ET-50 protocol. Selected exposure time was 3, 5 and 18 h. 1% triton-X-100 was used as a positive control, distilled water was used as a negative control [29]. 

Phototoxicity of the multiple emulsion was evaluated according to the pre-validated protocol [56,57]. For the exposure, five different concentrations of the multiple emulsion were topically applied on the 3D reconstructed human epidermis tissue and after the incubation exposed to 6 J/cm^3^ UVA. Chlorpromazine solution was used as positive control, distilled water as negative control [57]. Three replicates of tissues were used for each experiment. At the end of incubation time skin cells viability was tested using the MTT (3-(4,5-dimethylthiazol-2-yl)-2,5-diphenyltetrazolium bromide) method. 

The inflammation marker IL-1α/IL-1F1 was determined by the Quantikine^®^ Human IL-1α/IL-1F1 immunoassay according to the DLA-50 protocol: 50 µL of assay diluent and 200 µL of the assay media obtained during in vitro skin irritation test after 3, 5 and 18 hours of exposure. The IL-1α concentration was determined by microplate spectrophotometer at 450 nm wavelength with a correction of 570 nm. The concentration was calculated using a standard curve (R^2^ = 0.99864).

### 3.8. The Measurement of Human Skin Moisture, Sebum Content, Pigmentation and Pore Size In Vivo

The controlled randomized trial was conducted according to the protocol of biomedical study “The effects of semisolid preparations (creams, gels) on human skin moisture, lipid content, pigmentation and skin pore size” (BE-2-26) issued by Lithuanian Bioethics Committee and by permission of the State Data Protection Inspectorate. The inclusion criteria were: healthy volunteers, speaking and understanding Lithuanian, age above 18 years old, without a medical history of skin conditions or diseases (dermatitis, psoriasis and etc.). The exclusion criteria were: younger than 18 years old, not speaking or understanding Lithuanian, a history of skin conditions or diseases. The in vivo study was carried out in an examination room under constant environmental conditions of 25 ± 2 °C temperature with 40–65% relative humidity. Volunteers signed the Terms of Informed Consent after being informed about the objectives and methods of the research. The participants were informed not to use any skin care products 12 h before and during the study. The experiment consisted of three steps:

1. The initial data collection: skin moisture, sebum content, pigmentation, pore size (clean skin, without any skin care product applied).
2. Double and multiple emulsions application and evaluation of their influence on skin moisture content after 15, 30, 60, 120, 180, 240 min. Emulsions of three different compositions were applied on the inner forearm of human volunteers (Table 2).
3. Evaluation of the influence of three different emulsion compositions on the skin moisture, sebum content, pigmentation and pore size after 7, 14, 21 and 28 days of daily use evaluation.

Approximately 300 µL of the sample was applied to a 5 cm^2^ skin area of the inner forearm. The obtained data were analyzed using “Skinsys” software. Determination of the hydration state of stratum corneum is generally based on electrical measurements on the skin surface based on the capacitance method [37]. Skin moisture content was determined using a corneometer MY808S (Scalar, Tokyo, Japan): the head of corneometer was pressed to the skin and held for 2–3 s.

Sebum content in basal skin layer was determined by sebumometric strips. The strips were pressed to the skin and held for couple seconds. The released amount of sebum was determined by a dermatoscopic camera Coscam CCL-215USB (Sometech, Seoul, Korea) equipped with 50× vertical illuminating cap. Skin pore size was measured using 100× side illuminating cap attached to the dermatoscopic camera. Skin pore size was expressed as mean ± SD.

Pigmentation in dermis layer was determined using a cosmetic camera equipped with 14× polarized filter cap.

### 3.9. Statistical Analysis

Data are presented as mean ± SD. Statistical analysis was performed by one-way and two-way analysis of variance (ANOVA), followed by Dunnett’s post test using the software package Prism v. 5.04 (GraphPad Software Inc., La Jolla, CA, USA). A value of *p* < 0.01 has been taken as the level of significance. 

## 4. Conclusions

Based on the obtained results, it can be concluded that the innovative multiple emulsion with herbal extracts (0.5% emulsifier ViscOptima SE, 20% olive oil, 7.5% ethanolic rosemary extract, 24.18% linseed extract, 44.03% oat extract) does not cause skin dryness, despite the fact that it contains ethanol, and when used daily, it moisturizes the skin and reduces pigmentation more effectively than binary control emulsions. The developed product can be used for skin care or as a semisolid pharmaceutical base to which lipophilic and hydrophilic active compounds can be added. However, additional studies should be carried out to evaluate the effect of the plant extracts, which were used to form and stabilize emulsion, on the moisture, sebum content and skin pigmentation in vivo.

## Figures and Tables

**Table 1:** Phytochemical composition of multiple emulsion.

Origin of Active Ingredients	Active Ingredients Found in W/O/W Emulsion	Amount (mg/g ± SD)
Rosemary extract	Rosmarinic acid (RA)	0.047 ± 0.001
	Oleanolic acid (OA)	0.028 ± 0.001
	Ursolic acid (UA)	0.053 ± 0.001
Linseed and oat extracts	l-rhamnose	0.795 ± 0.001
	l-(+)-arabinose	269.810 ± 0.005
	d-(+)-xylose	4.80 ± 0.01
	d-(+)-galacturonic acid	3.02 ± 0.02
Oat extract	β-glucan	1.70 ± 0.01

**Table 2:** The composition of multiple and double emulsions.

Emulsion Phase	Investigated Samples					
	O/W Type Emulsion	(%)	O/W Type Emulsion Containing Ethanol	(%)	W/O/W Emulsion	(%)
Lipophilic phase	Olive oil	20	Olive oil	20	Olive oil	20
	Viscoptima SE	0.5	Viscoptima SE	0.5	Viscoptima SE	0.5
Hydrophilic phase	Distilled water	up to 100	Ethanol (90%)	7.5	Ethanolic rosemary extract	7.5
					Linseed extract	24.18
			Distilled water	up to 100	Oat extract	44.03
					Distilled water	up to 100

**Table 3:** The influence of the investigated emulsions on the skin pore size.

Time (days)	Investigated Samples		
	O/W Type Emulsion	O/W type Emulsion Containing Ethanol	W/O/W Type Emulsion
	Pore Size (mm)		
0	0.0326 ± 0.0035	0.0302 ± 0.0011	0.0335 ± 0.0032
7	0.0340 ± 0.0033	0.0324 ± 0.0013 *	0.0329 ± 0.0025
14	0.0352 ± 0.0024	0.0356 ± 0.0024 *	0.0324 ± 0.0031
21	0.0341 ± 0.0031	0.0355 ± 0.0035 *	0.032 1± 0.0012
28	0.0350 ± 0.0011	0.0350 ± 0.0034 *	0.0319 ± 0.0029

* *p* < 0.01 vs. 0 days. N = 42 volunteers

## References

[1] Henriksen L., Simonsen J., Haerskjold A., Linder M., Kieler H., Thomsen S.F., Stensballe L.G. (2015). Incidence rates of atopic dermatitis, asthma, and allergic rhinoconjunctivitis in Danish and Swedish children. J. Allergy Clin. Immunol..

[2] Zhu Y.-P., Woerdenbag H.J. (1995). Traditional Chinese herbal medicine. Pharm. World Sci..

[3] Choochote W., Chaiyasit D., Kanjanapothi D., Rattanachanpichai E., Jitpakdi A., Tuetun B., Pitasawat B. (2005). Chemical composition and anti-mosquito potential of rhizome extract and volatile oil derived from *Curcuma aromatica* against *Aedes aegypti* (Diptera: Culicidae). J. Vector. Ecol..

[4] Astier C., Benchad Y., Moneret-Vautrin D.A., Bihain B., Kanny G. (2010). Anaphylaxis to argan oil. Allergy.

[5] Ortiz K.J., Yiannias J.A. (2004). Contact dermatitis to cosmetics, fragrances, and botanicals. Dermatol. Ther..

[6] Kiken D.A., Cohen D.E. (2002). Contact dermatitis to botanical extracts. Am. J. Contact Dermat..

[7] Pigatto P., Bigardi A., Caputo R., Angelini G., Foti C., Grandolfo M., Rizer R.L. (1997). An evaluation of the allergic contact dermatitis potential of colloidal grain suspensions. Am. J. Contact Derm..

[8] Yamakawa Y., Ohsuna H., Aihara M., Tsubaki K., Ikezawa Z. (2001). Contact urticaria from rice. Contact Derm..

[9] Amaro C., Goossens A. (2008). Immunological occupational contact urticaria and contact dermatitis from proteins: A review. Contact Derm..

[10] Cizauskaite U., Ivanauskas L., Jakštas V., Marksiene R., Jonaitiene L., Bernatoniene J. (2016). *Rosmarinus officinalis* L. extract and some of its active ingredients as potential emulsion stabilizers: A new approach to the formation of multiple (W/O/W) emulsion. Pharm. Dev. Technol..

[11] Cizauskaite U., Marksiene R., Viliene V., Gruzauskas R., Bernatoniene J. (2017). New strategy of multiple emulsion formation based on the interactions between polymeric emulsifier and natural ingredients. Colloids Surf. A Physicochem. Eng. Asp..

[12] Khan A.Y., Talegaonkar S., Iqbal Z., Ahmed F.J., Khar R.K. (2006). Multiple emulsions: An overview. Curr. Drug Deliv..

[13] Kumar R., Kumar M.S., Mahadevan N. (2012). Multiple emulsions: A review. Int. J. Recent Adv. Pharm. Res..

[14] Bouyer E., Mekhloufi G., Rosilio V., Grossiord J.-L., Agnely F. (2012). Proteins, polysaccharides, and their complexes used as stabilizers for emulsions: Alternatives to synthetic surfactants in the pharmaceutical field?. Int. J. Pharm..

[15] Burgos-Díaz C., Rubilar M., Morales E., Medina C., Acevedo F., Marqués A.M., Shene C. (2016). Naturally occurring protein–polysaccharide complexes from linseed (*Linum usitatissimum*) as bioemulsifiers. Eur. J. Lipid Sci. Technol..

[16] Fuchs S.M., Schliemann-Willers S., Fischer T.W., Elsner P. (2005). Protective Effects of Different Marigold (*Calendula officinalis* L.) and Rosemary Cream Preparations against Sodium-Lauryl-Sulfate-Induced Irritant Contact Dermatitis. Skin Pharmacol. Phys..

[17] Stingni L., Lapomarda V., Lisi P. (1995). Occupational hand dermatitis in hospital environments. Contact Derm..

[18] Rasul A., Akhtar N. (2011). Formulation and in vivo evaluation for anti-aging effects of an emulsion containing basil extract using non-invasive biophysical techniques. DARU: J. Fac. Pharm. Tehran Univ. Med. Sci..

[19] Montenegro L., Pasquinucci L., Zappalà A., Chiechio S., Turnaturi R., Parenti C. (2017). Rosemary Essential Oil-Loaded Lipid Nanoparticles: In Vivo Topical Activity from Gel Vehicles. Pharmaceutics.

[20] Park M., Han J., Lee C.S., Heung Soo B., Lim K.M., Ha H. (2013). Carnosic acid, a phenolic diterpene from rosemary, prevents UV-induced expression of matrix metalloproteinases in human skin fibroblasts and keratinocytes. Exp. Dermatol..

[21] Vie K., Cours-Darne S., Vienne M., Boyer F., Fabre B., Dupuy P. (2002). Modulating effects of oatmeal extracts in the sodium lauryl sulfate skin irritancy model. Skin Pharmacol. Phys..

[22] Michelle Garay M. (2016). Colloidal Oatmeal (*Avena Sativa*) Improves Skin Barrier Through Multi-Therapy Activity. J. Drugs Dermatol..

[23] Criquet M., Roure R., Dayan L., Nollent V., Bertin C. (2012). Safety and efficacy of personal care products containing colloidal oatmeal. Clin. Cosmet. Investig. Dermatol..

[24] Goyal A., Sharma V., Upadhyay N., Gill S., Sihag M. (2014). Flax and flaxseed oil: An ancient medicine & modern functional food. J. Food Sci. Technol..

[25] Péterszegi G., Isnard N., Robert A., Robert L. (2003). Studies on skin aging. Preparation and properties of fucose-rich oligo-and polysaccharides. Effect on fibroblast proliferation and survival. Biomed. Pharmacother..

[26] Cizauskaite U., Marksa M., Bernatoniene J. (2018). The optimization of technological processes, stability and microbiological evaluation of innovative natural ingredients-based multiple emulsion. Pharm. Dev. Technol..

[27] Coecke S., Pelkonen O., Leite S.B., Bernauer U., Bessems J.G., Bois F.Y., Gundert-Remy U., Loizou G., Testai E., Zaldívar J.-M. (2013). Toxicokinetics as a key to the integrated toxicity risk assessment based primarily on non-animal approaches. Toxicol. In Vitro.

[28] Liebsch M., Barrabas C., Traue D., Spielmann H. (1997). Development of a new in vitro test for dermal phototoxicity using a model of reconstituted human epidermis. Altex.

[29] Kandárová H., Liebsch M., Genschow E., Gerner I., Traue D., Slawik B., Spielmann H. (2004). Optimisation of the EpiDerm test protocol for the upcoming ECVAM validation study on in vitro skin irritation tests. Altex.

[30] Buzek J., Ask B. (2009). Regulation (EC) No 1223/2009 of the European Parliament and of the Council of 30 November 2009 on cosmetic products. Off. J. Eur. Union L.

[31] Vecino X., Cruz J.M., Moldes A.B., Rodrigues L.R. (2017). Biosurfactants in cosmetic formulations: Trends and challenges. Crit. Rev. Biotechnol..

[32] Evy P. (2002). Contact sensitization from Compositae-containing herbal remedies and cosmetics. Contact Derm..

[33] Bárány E., Lindberg M., Lodén M. (2000). Unexpected skin barrier influence from nonionic emulsifiers. Int. J. Pharm..

[34] Guin J.D. (2005). Use of consumer product ingredients for patch testing. Dermatitis.

[35] Tornier C., Rosdy M., Maibach H.I. (2006). In vitro skin irritation testing on reconstituted human epidermis: Reproducibility for 50 chemicals tested with two protocols. Toxicol. In Vitro.

[36] Ferreira A., Vecino X., Ferreira D., Cruz J.M., Moldes A.B., Rodrigues L.R. (2017). Novel cosmetic formulations containing a biosurfactant from *Lactobacillus paracasei*.. Coll. Surf. B Biointerfaces.

[37] Yapar E.A., İNAL Ö., Erdal M.S. (2013). Design and in vivo evaluation of emulgel formulations including green tea extract and rose oil. Acta Pharm..

[38] Otto A., Du Plessis J., Wiechers J.W. (2009). Formulation effects of topical emulsions on transdermal and dermal delivery. Int. J. Cosmet. Sci..

[39] Pershing L.K., Lambert L.D., Knutson K. (1990). Mechanism of ethanol-enhanced estradiol permeation across human skin in vivo. Pharm. Res..

[40] Pecquet C., Pradalier A., Dry J. (1992). Allergic contact dermatitis from ethanol in a transdermal estradiol patch. Contact Derm..

[41] Pietsch H. (2001). Hand antiseptics: Rubs versus scrubs, alcoholic solutions versus alcoholic gels. J. Hosp. Infect..

[42] Flament F., Francois G., Qiu H., Ye C., Hanaya T., Batisse D., Cointereau-Chardon S., Seixas M.D.G., Dal Belo S.E., Bazin R. (2015). Facial skin pores: A multiethnic study. Clin. Cosmet. Investig. Dermatol.

[43] Roh M., Han M., Kim D., Chung K. (2006). Sebum output as a factor contributing to the size of facial pores. Br. J. Dermatol..

[44] Duffy J.A., Znaiden A.P. (1995). Composition and method for visibly reducing the size of skin pores. U.S. Patent.

[45] Kramer A., Bernig T., Kampf G. (2002). Clinical double-blind trial on the dermal tolerance and user acceptability of six alcohol-based hand disinfectants for hygienic hand disinfection. J. Hosp. Infect..

[46] Akhtar N., Yazan Y. (2008). Formulation and in vivo evaluation of a cosmetic multiple emulsion containing vitamin C and wheat protein. Pak. J. Pharm. Sci..

[47] Tran T.-N. T., Schulman J., Fisher D.E. (2008). UV and pigmentation: Molecular mechanisms and social controversies. Pigment Cell. Melanoma Res..

[48] Friedmann P.S., Gilchrest B.A. (1987). Ultraviolet radiation directly induces pigment production by cultured human melanocytes. J. Cell. Physiol..

[49] Nobile V., Michelotti A., Cestone E., Caturla N., Castillo J., Benavente-García O., Pérez-Sánchez A., Micol V. (2016). Skin photoprotective and antiageing effects of a combination of rosemary (*Rosmarinus officinalis*) and grapefruit (*Citrus paradisi*) polyphenols. Food Nutr. Res..

[50] Martin R., Pierrard C., Lejeune F., Hilaire P., Breton L., Bernerd F. (2008). Photoprotective effect of a water-soluble extract of *Rosmarinus officinalis* L. against UV-induced matrix metalloproteinase-1 in human dermal fibroblasts and reconstructed skin. Eur. J. Dermatol..

[51] Sánchez-Campillo M., Gabaldon J., Castillo J., Benavente-García O., Del Baño M., Alcaraz M., Vicente V., Alvarez N., Lozano J. (2009). Rosmarinic acid, a photo-protective agent against UV and other ionizing radiations. Food Chem. Toxicol..

[52] Suggs A., Oyetakin-White P., Baron D.E. (2014). Effect of botanicals on inflammation and skin aging: Analyzing the evidence. Inflamm. Allergy Drug Targets.

[53] Offord E.A., Gautier J.-C., Avanti O., Scaletta C., Runge F., Krämer K., Applegate L.A. (2002). Photoprotective potential of lycopene, β-carotene, vitamin E, vitamin C and carnosic acid in UVA-irradiated human skin fibroblasts. Free Radic. Biol. Med..

[54] McCleary B.V., Codd R. (1991). Measurement of (1→3),(1→4)-β-d-glucan in barley and oats: A streamlined enzymic procedure. J. Sci. Food Agric..

[55] Emaga T.H., Rabetafika N., Blecker C.S., Paquot M. (2012). Kinetics of the hydrolysis of polysaccharide galacturonic acid and neutral sugars chains from flaxseed mucilage. Biotechnology.

[56] Spielmann H., Müller L., Averbeck D., Balls M., Brendler-Schwaab S., Castell J.V., Curren R., De Silva O., Gibbs N., Liebsch M. (2000). The second ECVAM workshop on phototoxicity testing. The report and recommendations of ECVAM workshop. Altern. Lab. Anim..

[57] Jones P.A., Lovell W.W., King A.V., Earl L.K. (2001). In vitro testing for phototoxic potential using the EpiDerm 3-D reconstructed human skin model. Toxicol. Mech. Methods.

